# Association of gut microbiota with the pathogenesis of SARS-CoV-2 Infection in people living with HIV

**DOI:** 10.1186/s12866-023-03157-5

**Published:** 2024-01-03

**Authors:** Aya Ishizaka, Michiko Koga, Taketoshi Mizutani, Seiya Yamayoshi, Kiyoko Iwatsuki-Horimoto, Eisuke Adachi, Yutaka Suzuki, Yoshihiro Kawaoka, Hiroshi Yotsuyanagi

**Affiliations:** 1 Division of Infectious Diseases, Advanced Clinical Research Center, the Institute of Medical Science, the University of Tokyo, Tokyo, Japan; 2 Department of Computational Biology and Medical Sciences, Graduate School of Frontier Sciences, the University of Tokyo, Chiba, Japan; 3 Division of Virology, Department of Microbiology and Immunology, Institute of Medical Science, The University of Tokyo, Tokyo, Japan; 4 The Research Center for Global Viral Diseases, National Center for Global Health and Medicine Research Institute, Tokyo, Japan; 5 Department of Infectious Diseases and Applied Immunology, IMSUT Hospital of Institute of Medical Science, the University of Tokyo, Tokyo, Japan; 6 Influenza Research Institute, Department of Pathobiological Sciences, School of Veterinary Medicine, University of Wisconsin–Madison, Madison, WI USA; 7 Pandemic Preparedness, Infection and Advanced Research Center, The University of Tokyo, Tokyo, Japan; 8 Department of Computational Biology and Medical Sciences, Graduate School of Frontier Sciences, the University of Tokyo, 6-2-3 Kashiwanoha, 277-0882 Kashiwa-shi, Chiba Japan; 9 Division of Infectious Diseases, Advanced Clinical Research Center, Institute of Medical Science, the University of Tokyo, 4-6-1 Shirokanedai, Minato-ku, 108-8639 Tokyo, Japan

**Keywords:** SARS-CoV-2, COVID-19, HIV, Microbiota, Post-acute COVID-19 syndrome

## Abstract

**Background:**

People living with HIV (PLWH) with chronic inflammation may have an increasing risk for coronavirus disease 2019 (COVID-19) severity; however, the impact of their gut microbiota on COVID-19 is not fully elucidated. Here, we analyzed the temporal changes in the gut microbiota composition of hospitalized severe acute respiratory syndrome coronavirus 2 (SARS-CoV-2)-infected PLWH (PLWH-CoV) and their correlation with COVID-19 severity.

**Result:**

The 16S rRNA analysis results using stool samples (along the timeline from disease onset) from 12 hospitalized PLWH-CoV, whose median CD4 + T cell count was 671 cells/µl, were compared to those of 19 healthy people and 25 PLWH. Bacterial diversity in PLWH-CoV is not significantly different from that of healthy people and SARS-CoV-2 non-infected PLWH, but a significant difference in the microbiota diversity was observed in the classification according to the disease severity. Immediately after the disease onset, remarkable changes were observed in the gut microbiota of PLWH-CoV, and the changing with a decrease in some short-chain fatty acid-producing bacteria and an increase in colitis-related pathobiont. In the second week after disease onset, relative amounts of specific bacteria distinguished between disease severity. One month after the disease onset, dysbiosis of the gut microbiota persisted, and the number of Enterobacteriaceae, mainly *Escherichia-Shigella*, which is potentially pathogenic, increased and were enriched in patients who developed post-acute sequelae of COVID-19 (PASC).

**Conclusion:**

The changes in the gut microbiota associated with SARS-CoV-2 infection observed in PLWH in this study indicated a persistent decrease in SCFA-producing bacteria and an intestinal environment with an increase in opportunistic pathogens associated with enteritis. This report demonstrates that the intestinal environment in PLWH tends to show delayed improvement even after COVID-19 recovery, and highlights the importance of the dysbiosis associated with SARS-CoV-2 infection as a potential factor in the COVID-19 severity and the PASC in PLWH.

**Supplementary Information:**

The online version contains supplementary material available at 10.1186/s12866-023-03157-5.

## Introduction

It has been argued that individuals with underlying disease are at increased risk for severe COVID-19 infection, but medical evidence of these findings is limited, and it would be beneficial to determine the causal relationship between immune status and disease progression [1]. Despite the effectiveness of antiretroviral therapy (ART), people living with HIV (PLWH) who are prone to underlying diseases, continue to have a chronic inflammatory state [2]. Although the reported susceptibility of PLWH to SARS-CoV-2 infection does not differ from that of the general population, as a matter of concern, PLWH are at risk for more severe COVID-19 disease due to immunological vulnerability and comorbidities [3]. Therefore, understanding of the link between SARS-CoV-2 infection and specific clinical and immunologic factors in PLWH is one of the significant issues. Early studies reported that, in the early stages of the pandemic among patients hospitalized with COVID-19, PLWH were at a higher risk of death [4, 5]. PLWH were more likely to be admitted to the ICU [6], and HIV infection was also an independent risk factor for severe COVID-19 and in-hospital death [7]. Conversely, comparative studies between PLWH and the general population have reported no significant changes in disease status [8–10], indicating that the severity of COVID-19 in PLWH also depends on comorbidities [11]. A recent meta-analysis also reported no difference in the risk of death between PLWH and HIV-seronegative individuals [12]. The further analysis reported inadequate viral immunological control as an underlying reason for COVID-19 severity and death in PLWH [13]. In summary, age, comorbidities, and lack of viral immunological control are risk factors for poor clinical outcomes of COVID-19 in PLWH, and it is important to determine the background of each patient [14, 15].

In recent years, an increasing number of reports have shown a relationship between the gut microbiota and pathological conditions, and it has become clear that the gut microbiota influences host homeostasis mechanisms, such as nutrition, metabolism, and immune regulation, and is associated with the progression of various pathological conditions [16]. A recent report indicates that the commensal microbiota influences the immune response after mRNA vaccination against SARS-CoV-2 [17, 18]. HIV infection is no exception, and infection-induced changes in the gut microbiota (dysbiosis) have been reported [19–22]. The changes are not only limited to the pathogenesis of HIV infection but are also indicated to affect host homeostasis [23].

Generally, there are two major trends in the intestinal microbiota of PLWH. The first is related to sexual practices and is unrelated to HIV infection. It is typically seen in men who have sex with men (MSM), who constitute a large portion of PLWH, regardless of their HIV infection status. This pattern is characterized by an increased amount of *Prevotella* spp. and decreased amount of *Bacteroides* spp., respectively [24–26]. Furthermore, HIV-induced gut microbiota abnormalities are associated with decreased alpha diversity and increased Gammaproteobacteria, including the Enterobacteriaceae, regardless of the sex or sexual orientation of the individual [27]; the gut microbiota of PLWH has been reported to be similar to that of various chronic inflammatory diseases, including inflammatory bowel disease [28], suggesting a potential relationship between HIV-related gut microbiota abnormalities and chronic inflammation. In addition, several butyrate-producing bacteria belonging to the Lachnospiraceae and Ruminococcaceae are reduced in HIV-infected individuals, which may be associated with persistent impairment of intestinal barrier function and easier transfer of inflammatory microbial products into the body circulation [19]. In our previous analysis of Japanese PLWH, an increase of bacteria belonging to Negativicutes, Coriobacteriia, and Bacilli, and a decrease of bacteria belonging to Clostridia were observed at the class level, compared to the gut microbiota of healthy subjects. Furthermore, changes in these bacteria in PLWH were also correlated with the level of inflammation [20]. In the hepatitis A virus (HAV) epidemic that occurred in Japanese PLWH in 2018, it was observed that significant changes in the gut microbiota occurred after infection (dysbiosis) and there was continued dysbiosis and detection of HAV in the stool for a long time after healing. Thus, these findings suggest that the immunocompromised state is associated with dysbiosis and delayed recovery of the intestinal environment [29].

Despite effective viral control, chronic inflammation is a prominent feature of PLWH and is recognized as a risk factor for the development and progression of age-related complications [30, 31]. In recent years, it has been increasingly recognized that persistent chronic inflammation and dysbiosis have been implicated in disease progression, patients with inflammatory bowel disease have decreased amounts of short-chain fatty acid-producing bacteria, which are beneficial to the intestinal tract and host homeostasis. Furthermore, changes in the gut microbiota have been reported immediately after the onset of COVID-19, and these changes are related to the severity of the disease [32, 33]. The gut microbiome profile has also been reported to be potentially involved in post-acute sequelae of COVID-19 and this may be due to the susceptibility of the gut microbiota to pro-inflammatory cytokines as well as viral antigens [34, 35]. Several risk factors for COVID-19 severity, including comorbidities, have been demonstrated in immunocompromised patients, including PLWH, however little is known about the direct involvement of the gut microbiota. Men who have sex with men (MSM) are known to have unique bacterial microbiota patterns [24, 26, 27]. In this study, we hypothesized that bacterial microbiota profile and immunodeficiency syndromes are associated with the development of COVID-19 in PLWH, mainly MSM, and we investigate the possible influence of the bacterial microbiota profile on COVID-19 disease progression. The overall purpose of this study was thus to examine changes in the gut microbiota during treatment of SARS-CoV-2-infected PLWH as it relates to COVID-19 disease severity in PLWH on long-term viral suppression with ART.

## Results

### Clinical characteristics of study participants

 Stool samples were collected between March 2020 and June 2022 from 12 PLWH admitted to IMSUT Hospital with COVID-19 confirmed by SARS-CoV-2 RT-qPCR (Table 1). All patients enrolled in the study were infected with alpha, delta, omicron BA.1, or BA.2 variants. These viral variants were treated equally in bacterial microbiota analysis. The demographic and clinical characteristics of the patients included in the study are presented in Table 2. The non-COVID-19 control cohort comprised 25 PLWH (median age of 50 years [range: 26–65 years]) who attended the University of Tokyo, Institute of Medical Science Hospital and indicated their willingness to participate in the study. The healthy cohort (HC) was comprised of 19 adults (median age of 48 years [range: 25–63 years]) who provided stool samples and indicated their willingness to participate in the study at the University of Tokyo. The PLWH-CoV cohort comprised 12 men with a median age of 47 years (range: 26–60 years). In terms of severity classification, 7 (58.3%) patients were mildly ill, four (33.3%) were moderately ill, and one (8.3%) was severely ill, none of whom required forced ventilation.

**Table 1:** General characteristics of COVID-19 patients and their clinical presentation in HIV treatment

Patient ID	Variant	Age	Severity	Stool collection days from COVID-19 onset				COVID-19 treatment						PACS	ART regimen	Comorbidities	Vaccination times before SARS-CoV-2 infection
				**d1-7**	**d8-14**	**d30 ~ **		**Steroid use**	**Antibiotics use**	**medication used 1**	**medication used 2**	**medication used 3**	**PPI**	**symptoms**			
1	α	**51**	mild	d6				No	No	day10-14			No	No	TAF/FTC/BIC	HIV, dyslipidemia, depression	No
										Lopinavir, ritonavir							
2	α	27	moderate	d3	d10			No	No	day1-5			No	No	TAF/FTC/BIC	HIV, syphilis, asthma	No
										Favipiravir							
3	α	54	severe		d10			Yes	Yes	day7-11	day6-7	day7-12	day6-	No	ABC/3TC/DTG	HIV, diabetes, high blood pressure, dyslipidemia, hyperuricemia, cerebral infarction	No
										Remdesivir	Dexamethasone	methylprednisolone	Rabeprazole Sodium				
4	α	42	mild	d5	d10	d53		No	No	No			No	No	TAF/FTC/DTG	HIV	No
5	α	26	mild	d2	d9			No	No	No			No	No	TAF/FTC/BIC	HIV	No
6	α	51	moderate	d5	d11	d82		No	No	No			No	No	TAF/FTC/BIC	HIV, atopic dermatitis, uveitis, asthma, dyslipidemia, obesity	No
7	α	53	moderate	d5	d8	d48		No	No	No			No	No	TAF/FTC/RPV	HIV, asthma, high blood pressure	No
8	α	46	mild	d2	d11	d67	d382	No	No	day3-7			No	Yes	CABLA + RPVLA	HIV	No
										Remdesivir				hair loss			
9	α	42	mild	d4	d13	d44		Yes	No	day9-13			day9-18	No	3TC/DTG	HIV, Herpes zoster	No
										Dexamethasone			Esomeprazole Magnesium Hydrate				
10	delta	60	moderate		d9			Yes	No	day8-12	day8-13		day8-13	No	TAF/FTC/BIC	HIV	No
										Remdesivir	Dexamethasone		Esomeprazole Magnesium Hydrate				
11	BA.1	54	mild	d4	d11	d31	d128	No	No	day3-7			No	Yes	ABC/3TC/DTG	HIV, sinusitis, allergic rhinitis, knee osteoarthritis, fatty liver	1 (mRNA-1273)
										Molnupiravir				Fatigue, brain fog			
12	BA.2	54	mild	d4	d9	d85		No	No	day2			No	No	3TC/DTG	HI, Kaposi's sarcom, lacunar infarction, fatty liver, renal cyst	2 (BNT162b)
										Sotrovimab

**Table 2:** Overview of the demographic and clinical characteristics of participants in this study

	**Healthy controls (*n* = 19)**	**PLWH (*n* = 25)**	**PLWH-CoV (*n* = 12)**	**p**
Age	48 ± 10.19	50 ± 9.63	46.7 ± 10.76	ns
No. (%) males	19 (100)	25 (100)	12 (100)	ns
No. (%) of MSM		20 (80)	10 (83.3)	ns
No. (%) with viral loads < 50 copies/ml		25 (100)	12 (100)	ns
CD4 count (cells/μl)		539.72 ± 163.1	671.28 ± 243^a^	ns *p* = 0.13
Nadir CD4 count (cells/μl)		184.36 ± 123.1	154.2 ± 97.7	ns
Time on ART (mo)		105.92 ± 49.8	106 ± 44.5	ns
BMI		24.69 ± 3.6	27.5 ± 5.3	ns* p* = 0.41
No. (%) of SARS-CoV-2 vaccination	0	0	2 (16.67)	
COVID-19 severity; No.(%)				
mild			7 (58.3)	
moderate			4 (33.3)	
severe			1 (8.3)	
Medical history; No.(%)				
SARS-CoV-2 infection	0	0	0	
AIDS	0	5 (20)	2 (16.67)	ns
diabetes	0	0	1 (8.3)	ns
high blood pressure	0	3 (12)	2 (16.67)	ns

^a^before SARS-CoV-2 infection

**Fig. 1:**
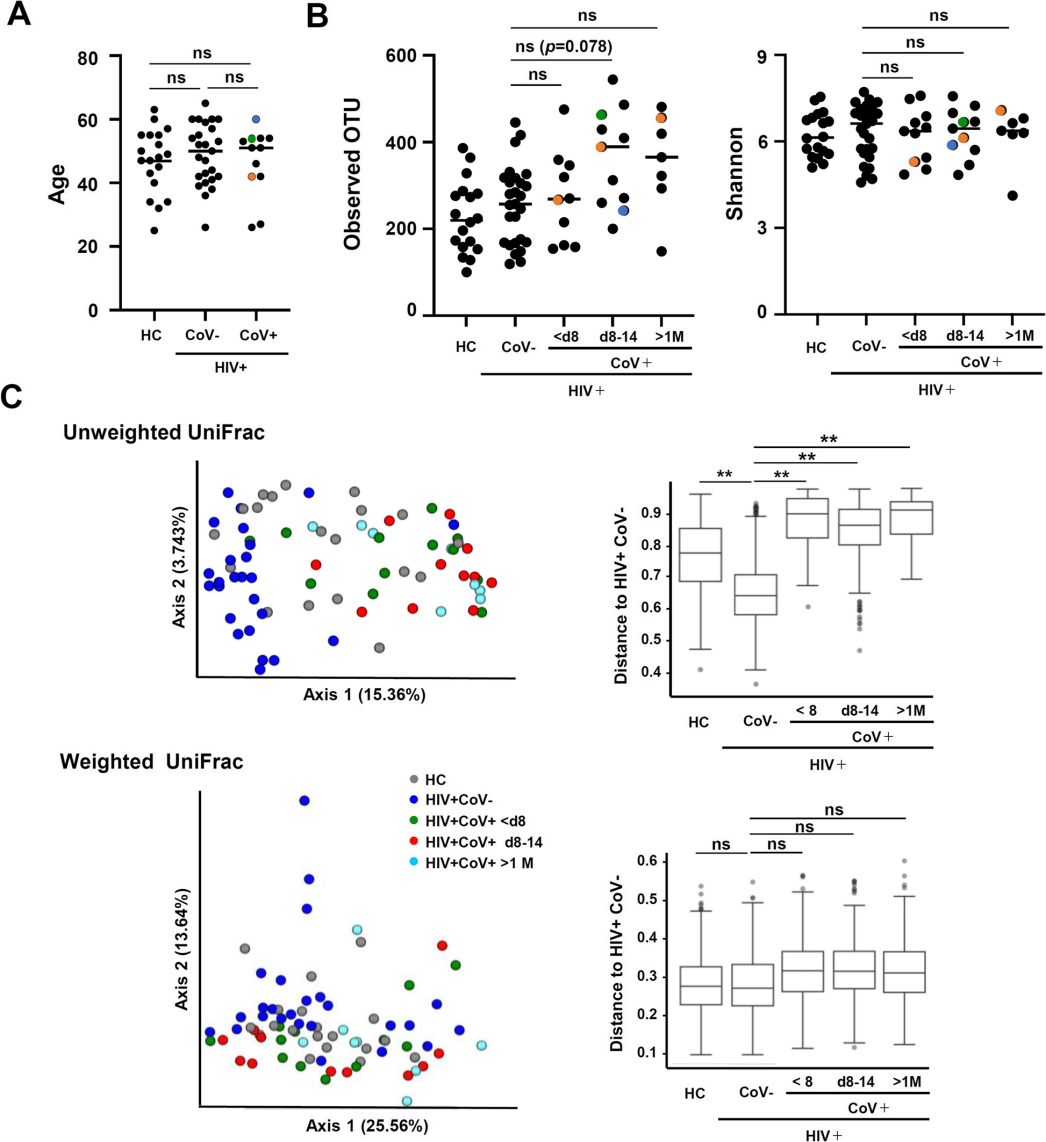
Temporal changes in the diversity of the gut microbiota after COVID-19 onset. **A**, **B** Age and diversity analysis of gut microbiota in the participants. Age of participants (**A**), Observed operational taxonomic units (OTUs) (left), Shannon analysis (right) (**B**), Points for patients who received steroids are shown in color (3 patients). **C** Beta diversity of gut microbiota of SARS-CoV-2 uninfected PLWH and infected PLWH. Principal coordinate analysis comparison by beta diversity values and comparison of the gut microbiota from individuals of PLWH and geographic locations of other SARS-CoV-2 infected PLWH (unweighted UniFrac distance to PLWH, top) and (weighted UniFrac distance to PLWH, bottom). HC; healthy control, ns: not significant, **p < 0.01, < d8: within 7 days of COVID-19 onset, d8-14: 8 − 14 days after COVID-19 onset, >1 M: over 1 month after COVID-19 onset

In the present analysis, PLWH and SARS-CoV-2-infected PLWH had received ART for over one year and had fewer than 20 copies/ml of HIV viral loads; 8 of the 12 PLWH infected with SARS-CoV-2 had comorbidities such as bronchial asthma, diabetes, and dyslipidemia (Table 1). All patients recovered and were discharged after an average hospital stay of 10 days (range, 6–14 days). Table 1 summarizes the treatments prescribed to each patient during hospitalization. During treatment for SARS-CoV-2 infection, three patients received steroids and proton pump inhibitors (PPI). There were no differences in age among the three groups (HC, PLWH, and PLWH-CoV) (Fig. 1A).

### Diversity analysis of the bacterial microbiota associated with SARS-CoV-2 Infection in PLWH

SARS-CoV-2 has been reported to cause changes in the gut microbiota of patients during the early stages of infection [32]. Therefore, to clarify the effects of SARS-CoV-2 infection on the gut microbiota of PLWH, we performed 16S rRNA sequence analysis of stool samples provided by PLWH infected with SARS-CoV-2 and hospitalized at the University of Tokyo, Institute of Medical Science Hospital. We previously reported that the gut microbiota of SARS-CoV-2-infected patients changed in the gut microbiota during hospitalization [32]. Therefore, to investigate changes in the gut microbiota from COVID-19 onset to recovery, stool samples obtained from PLWH-CoV patients were classified into three groups according to the collection date: within seven days of onset (< 8 days); 8–14 days; and one month later; and 16S rRNA sequencing was conducted. Then, a diversity analysis of the gut bacterial microbiota of HC, PLWH, and PLWH-CoV (< 8 days, 8–14 days, > 1 month) was performed. A comparison of Observed operational taxonomic unit (Observed OTU) showed no difference between HC and PLWH (Fig. 1B, left). In comparison to PLWH, an increase in Observed OTU values was observed from one week to one month after onset in PLWH-CoV, but no statistically significant differences were found between them. Shannon index values did not differ among the three groups (Fig. 1B, right). Principal Coordinate Analysis (PCoA) was then performed to compare the overall composition (beta diversity) of the microbial communities based on UniFrac distance measurements. In plots based on PCoA, HC and PLWH or PLWH and PLWH-CoV were relatively clearly separated in the unweighted analysis, which only considers account bacterial existence or absence, at each period from onset (< 8 days, 8–14 days, and > 1 month) (Fig. 1C top, Supplementary Table 1). However, the weighted uniFrac distance, which takes the abundance of observed organisms into account, showed no clear difference among them (Fig. 1C bottom, Supplementary Table 1).

### Changes in bacterial microbiota over time due to SARS-COV-2 Infection in PLWH

**Fig. 2:**
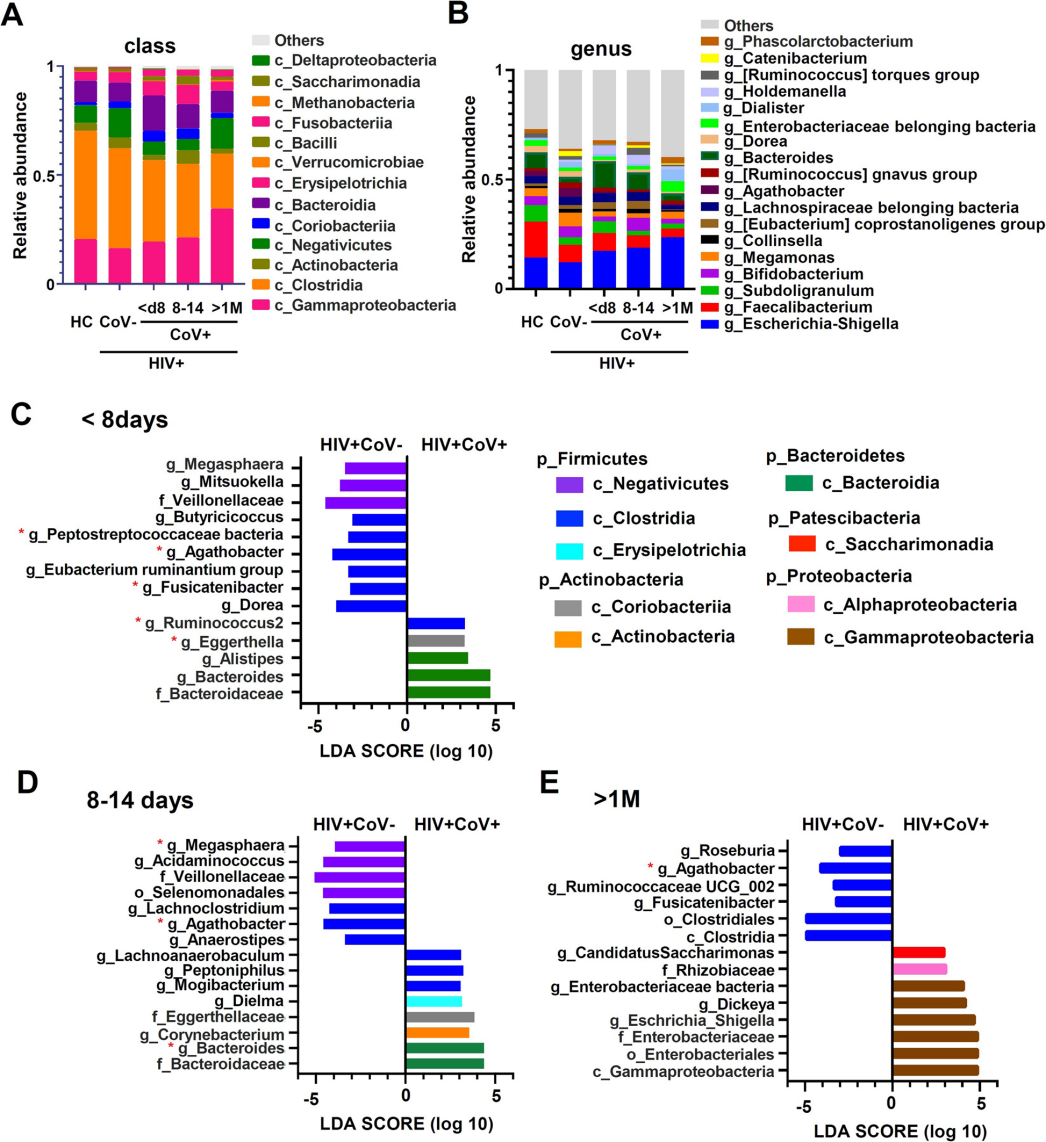
Changes in the gut microbiota in SARS-CoV-2-infected PLWH after COVID-19 onset. **A**, **B** Taxa bar plot for main class (**A**) or genus (**B**) of gut microbiota in participants. **C**-**E**)Changes in gut microbiota in SARS-CoV-2 infected PLWH were analyzed using linear discriminant analysis (LDA) effect size in comparison with the SARS-CoV-2 uninfected PLWH. < d8: within 7 days of COVID-19 onset (*n* = 10) (**C**), d8-14: 8 − 14 days after COVID-19 onset (*n* = 9) (**D**), and > 1 M: over 1 month after COVID-19 onset (*n* = 7) (**E**). Red asterisks (*) indicate bacterial genera with statistically significant differences in the multivariate analysis with linear models (MaAsLin)2 analysis adjusted for body mass index (BMI). All analysis was calculated with LDA > 3, p: phylum, c: class, o: order, f: family, g: genus

Next, to analyze how SARS-CoV-2 infection changes in the gut microbiota of PLWH based on the bacterial hierarchy, we classified the stool samples collected as one group every week from the onset date: within eight days (< 8 days), 8–14 days, and one month later (> 1 M) from the onset. At the class level, as an overall movement of the bacterial microbiota, we observed that the gut microbiota changed over time immediately after disease onset, with a gradual decrease in the Clostridia class (Fig. 2A). Conversely, the classes Gammaproteobacteria and Negativicutes gradually increased from onset to one month, and enrichment of the Bacteroidia class was observed in the early stages of disease onset. In contrast, the Erysipelotrichia class showed a peak at 8–14 days after onset. Furthermore, analysis at the genus level showed that *Faecalibacterium* and *Subdoligranulum*, which belong to the genus Clostridia, tended to be present in lower amounts in PLWH than in the HC, but were further reduced in PLWH-CoV after SARS-CoV-2 infection (Fig. 2B). *Escherichia-Shigella*, which belongs to the Gammaproteobacterial class, increased with time after the onset of illness, and *Bacteroides*, which belongs to the Bacteroidia class, was observed to be enriched immediately after onset.

 We compared temporal changes in the gut microbiota of PLWH-CoV and PLWH using (Linear discriminant analysis Effect Size) LEfSe analysis. Immediately after onset (< 8 days), PLWH-CoV showed a decrease in bacteria belonging mainly to the Clostridia and Negativicutes classes, and an increase in bacteria belonging to the Bacteroidia class (Fig. 2C). A similar trend was observed eight days after disease onset, with a decrease in bacteria belonging to the Negativicutes class and an increase in some bacteria belonging to the Clostridia and Erysipelotrichia classes (Fig. 2D). After discharge from the hospital, one month after the onset of the disease, the decrease in the Negativicutes class and the enrichment of the Bacteroidia class resolved, but a decrease in some bacteria belonging to the Clostridia class continued to be observed. In contrast, the enrichment of bacteria belonging to the Gammaproteobacteria, Alphaproteobacteria, and Saccharimonadia classes was observed in PLWH-CoV (Fig. 2E). Because body weight is related to metabolism and associated with changes in the intestinal microbiota changes [36], it was considered to be a potential confounder of the changes in the intestinal microbiota due to SARS-CoV-2 infection. Therefore, in the LEfSe analysis of changes in gut microbiota in SARS-CoV-2 infection, we adjusted for body mass index (BMI) using multivariate analysis with linear models (MaAsLin)2 (Table 3). Even after adjusting for BMI, the results showed a decrease in short chain fatty acid (SCFA)-producing bacteria, mainly *Agathobacter* (Fig. 2C-E; Table 3), and an increase in *Bacteroides* (Fig. 2D; Table 3).

**Table 3:** MaAsLin2 analysis of microbiological features associated with SARS-CoV-2 infection in PLWH

**< d8_significant_results**			
**feature**	**coefficient**	***p* value**	**q value**
k__Bacteria.p__Firmicutes.c__Clostridia.o__Clostridiales.f__Lachnospiraceae.g__Agathobacter	-3.529475	0.014136	0.232761
k__Bacteria.p__Firmicutes.c__Clostridia.o__Clostridiales.f__Peptostreptococcaceae.f__Peptostreptococcaceae.unassigned	-3.514981	0.001127	0.180114
k__Bacteria.p__Firmicutes.c__Clostridia.o__Clostridiales.f__Lachnospiraceae.g__Anaerostipes	-3.238904	0.006077	0.198141
k__Bacteria.p__Firmicutes.c__Clostridia.o__Clostridiales.f__Lachnospiraceae.g__Fusicatenibacter	-3.012233	0.00199	0.180114
k__Bacteria.p__Firmicutes.c__Clostridia.o__Clostridiales.f__Lachnospiraceae.g__.Ruminococcus..gauvreauii.group	-2.365259	0.009122	0.198141
k__Bacteria.p__Firmicutes.c__Negativicutes.o__Selenomonadales.f__Veillonellaceae.g__Allisonella	-2.218109	0.007567	0.198141
k__Bacteria.p__Proteobacteria.c__Gammaproteobacteria.o__Xanthomonadales.f__Xanthomonadaceae.g__Lysobacter	-2.012181	0.007466	0.198141
k__Bacteria.p__Firmicutes.c__Clostridia.o__Clostridiales.f__Lachnospiraceae.g__Lachnospiraceae.FCS020.group	-1.921816	0.008338	0.198141
k__Bacteria.p__Firmicutes.c__Clostridia.o__Clostridiales.f__Peptostreptococcaceae.g__Peptostreptococcus	0.881346	0.006571	0.198141
k__Bacteria.p__Firmicutes.c__Bacilli.o__Bacillales.f__Staphylococcaceae.g__Staphylococcus	1.153639	0.006334	0.198141
k__Bacteria.p__Proteobacteria.c__Gammaproteobacteria.o__Betaproteobacteriales.f__Burkholderiaceae.g__Ralstonia	1.225529	0.009374	0.198141
k__Bacteria.p__Proteobacteria.c__Gammaproteobacteria.o__Betaproteobacteriales.f__Burkholderiaceae.g__Burkholderia.Caballeronia.Paraburkholderia	1.342507	0.007972	0.198141
k__Bacteria.p__Firmicutes.c__Clostridia.o__Clostridiales.f__Eubacteriaceae.g__Anaerofustis	1.467882	0.013115	0.232761
k__Bacteria.p__Firmicutes.c__Clostridia.o__Clostridiales.f__Ruminococcaceae.g__Ruminococcus.2	1.991821	0.009673	0.198141
k__Bacteria.p__Firmicutes.c__Clostridia.o__Clostridiales.f__Ruminococcaceae.g__GCA.900066225	2.081854	0.004563	0.198141
k__Bacteria.p__Actinobacteria.c__Coriobacteriia.o__Coriobacteriales.f__Eggerthellaceae.g__Eggerthella	2.237567	0.009852	0.198141
k__Bacteria.p__Patescibacteria.c__Saccharimonadia.o__Saccharimonadales.f__Saccharimonadaceae.g__TM7.phylum.sp..oral.clone.DR034	2.85835	0.003307	0.198141
**d8-14_significant_results**			
**feature**	**coefficient**	***p* value**	**q value**
k__Bacteria.p__Firmicutes.c__Negativicutes.o__Selenomonadales.f__Veillonellaceae.g__Megasphaera	-4.9886951	0.0012297	0.1516661
k__Bacteria.p__Firmicutes.c__Clostridia.o__Clostridiales.f__Lachnospiraceae.g__Agathobacter	-4.6955635	0.0007094	0.1432304
k__Bacteria.p__Actinobacteria.c__Coriobacteriia.o__Coriobacteriales.f__Eggerthellaceae.g__Gordonibacter	0.7041275	0.0053219	0.1969091
k__Bacteria.p__Firmicutes.c__Clostridia.o__Clostridiales.f__Ruminococcaceae.g__GCA.900066225	1.931992	0.0007742	0.1432304
k__Bacteria.p__Bacteroidetes.c__Bacteroidia.o__Bacteroidales.f__Bacteroidaceae.g__Bacteroides	2.8077757	0.0052738	0.1969091
k__Bacteria.p__Firmicutes.c__Clostridia.o__Clostridiales.f__Eubacteriaceae.g__Eubacterium	3.2239335	0.0029386	0.1969091
** > 1M_significant_results**			
**feature**	**coefficient**	***p* value**	**q value**
k__Bacteria.p__Firmicutes.c__Clostridia.o__Clostridiales.f__Lachnospiraceae.g__Agathobacter	-5.6216565	0.0025782	0.2217262
k__Bacteria.p__Firmicutes.c__Clostridia.o__Clostridiales.f__Ruminococcaceae.g__GCA.900066225	1.7187556	0.0007951	0.1367651
k__Bacteria.p__Firmicutes.c__Clostridia.o__Clostridiales.f__Ruminococcaceae.g__Ruminococcus.2	1.2760845	0.002154	0.2217262

### Association of CD4 + T cell changes with pathophysiology and gut microbiota

**Fig. 3:**
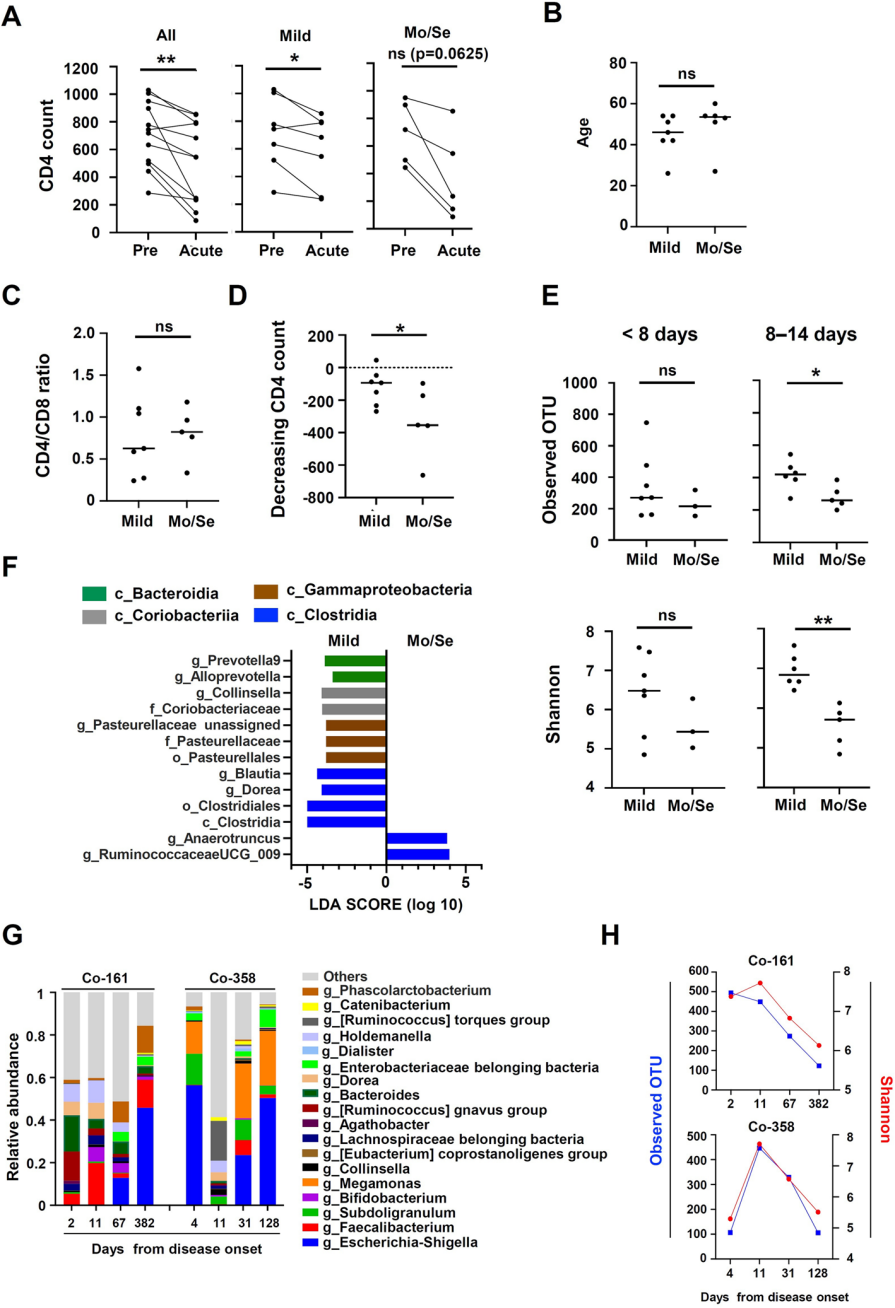
Correlation between the gut microbiota behavior and pathogenesis after SARS-CoV-2 infection in PLWH. (**A**) Comparison of plasma CD4 T-cell counts between PLWH-CoV patients with mild (*n* = 7) and moderate/severe (Mo/Se) (*n* = 5) COVID-19 disease before (Pre) and after (Acute) SARS-CoV-2 infection (**B**–**D**) Comparison of age (**B**), CD4/CD8 ratio (**C**) and CD4 depletion numbers (**D**) in patients with PLWH-CoV according to disease severity (mild, *n* = 7 vs. Mo/Se, *n* = 5). **E** Diversity analysis in PLWH-CoV patients classified by disease severity. Observed OTUs (top) and Shannon index (bottom). <d8, mild (*n* = 7), Mo/Se (*n* = 3), d8-14, mild (*n* = 6), Mo/Se (*n* = 5) (**F**) Changes in the gut microbiota in PLWH-CoV (d8-14) were analyzed by linear discriminant analysis (LDA) effect size, compared by disease severity (mild, *n* = 6 vs. Mo/Se, *n* = 5). **G** and **H** Longitudinal changing of gut microbiota for main genus of gut microbiota (**G**) and Longitudinal plots of Observed OTUs (blue) and Shannon index (red) (**H**) in Co-161 and Co-358 who had PACS symptoms. ns not significant, *p < 0.05, **p < 0.01, c: class, o: order, f: family, g: genus

Since it has been reported that SARS-CoV-2 infection decreases CD4 + T cell count [37], we performed a comparative analysis of CD4 + T cell levels and pathology in PLWH infected with SARS-CoV-2. Changes in the CD4 + T cell count and plasma HIV viral load from one year before infection to 6 months after are shown in Supplementary Fig. 1. In this study, the participants experienced a decrease in their CD4 + T cell level immediately after the onset of SARS-CoV-2 infection, but did not experience a change in their HIV viral load (Fig. 3A, left and Supplementary Fig. 1). We analyzed the change in CD4 + T cell levels after SARS-CoV-2 infection, dividing the patients into two groups: a mild disease group and a moderate or severe (Mo/Se) disease group (Supplemental Table 2). The mild disease group experienced a statistically significant decrease in their CD4 + T cell levels (Fig. 3A, middle). In the Mo/Se disease group, the decrease in each CD4 + T cell levels were greater than those in mild disease group but not statistically significant (Fig. 3A, right). Older individuals are at higher risk of developing severe COVID-19 [14, 15]. In this study, older age was more prevalent in the Mo/Se group although this association was not statistically significant (Fig. 3B). The presence of comorbidities was moderately associated with COVID-19 disease severity, with one of five PLWH with no comorbidities, and four of seven PLWH with comorbidities, having Mo/Se disease (Table 1). The CD4/CD8 ratio, which reflects the immunological condition, measured in the most recent period of infection (within 6 months before infection) was not associated with disease severity (Fig. 3C). In addition, a comparison of the decrease in CD4 + T cell levels before and after infection showed that the Mo/Se group had a significantly greater decrease in CD4 + T cell levels than those in the mild disease group (Fig. 3D). Furthermore, because no significant differences were observed in the medical backgrounds of the mild and the Mo/Se groups (Supplementary Table 2), the diversity of the gut microbiota was analyzed according to disease severity using the Mann-Whitney U test. The patients in the mild group had a significantly greater increase in Observed OTU (*p* = 0.017) and Shannon index (*p* = 0.0043) in the second week after the onset of COVID-19 than those in the Mo/Se disease group (Fig. 3E). As differences were observed in the diversity of the bacterial microbiota 8–14 days after the onset of SARS-CoV-2 infection, we classified patients by the severity of their illness on days 8–14, and analyzed differences in bacterial microbiota profiles again using LEfSe. The counts of *Prevotella 9* and *Alloprevotella* belonging to the class Bacteroidia, and *Collinsella* belonging to the class Coriobacteriia, were lower in the Mo/Se group than in the mild disease group. The counts of *Dorea* and *Blautia*, which belong to the Clostridia class, and their dependents belonging to Pasteurellales order or the Gammaproteobacteria class, were also decreased (Fig. 3F). At this time, the decrease in the certain genera (*Dorea*, *Blautia*, *Prevotella 9*, *Alloprevotella* and *Collinsella*) was more pronounced in the group without comorbidities than in the group with comorbidities. (Supplementary Fig. 2A). In the Mo/Se group, a decreasing trend of short-chain fatty acid (SCFA)-producing bacteria was observed from the first to the second week after the onset of the disease compared to the mild disease group. While an increase was observed in *Escherichia-Shigella* in the Mo/Se group (Supplementary Fig. 3). In contrast, a part of the class Clostridia belonging bacteria *such as Anaerotruncus* and *Ruminococcaceae UCG_009* were enriched in patients in the Mo/Se group (Fig. 3F). Since it has also been reported that patient polypharmacy may contribute to COVID-19 severity in PLWH [3], we compared the duration of previous treatment in 12 patients but found no correlation between the severity of disease and duration of treatment (Supplementary Fig. 2B).

### Association of post-acute COVID-19 syndromes with the gut microbiome

Post-acute sequelae of COVID-19 (PASC) have been reported to be present in approximately 30% of PLWH 6 months after the onset of COVID-19 [38]. In this study, 7 of the 12 patients provided stool specimens after discharge from the hospital, and 2 (17%) had subjective symptoms of PASC (Table 1). In these two patients, we analyzed the changes in the gut microbiota over time up to 128 and 382 days after onset (Fig. 3G). Both patients had an enrichment of Enterobacteriaceae and its dependent bacteria, *Escherichia*-*Shigella*, were still present 4 months after the onset, whereas a decrease in *Bifidobacterium* was observed. In addition, enrichment of *Phascolarctobacterium* and *Megamonas* was observed mainly in patients Co-161 and Co-358, respectively. A decrease in the diversity of the bacterial microbiota was observed after a transient increase during the acute phase after the disease onset (Fig. 3H). In contrast, 4 of the 5 patients who did not experience PASC symptoms did not experience a decrease in diversity of their bacterial microbiota (observed OTU, Shannon index) (Supplementary Fig. 2C).

### SARS-CoV-2 Infection induces increased bacterial metabolic functions in PLWH-CoV

**Fig. 4:**
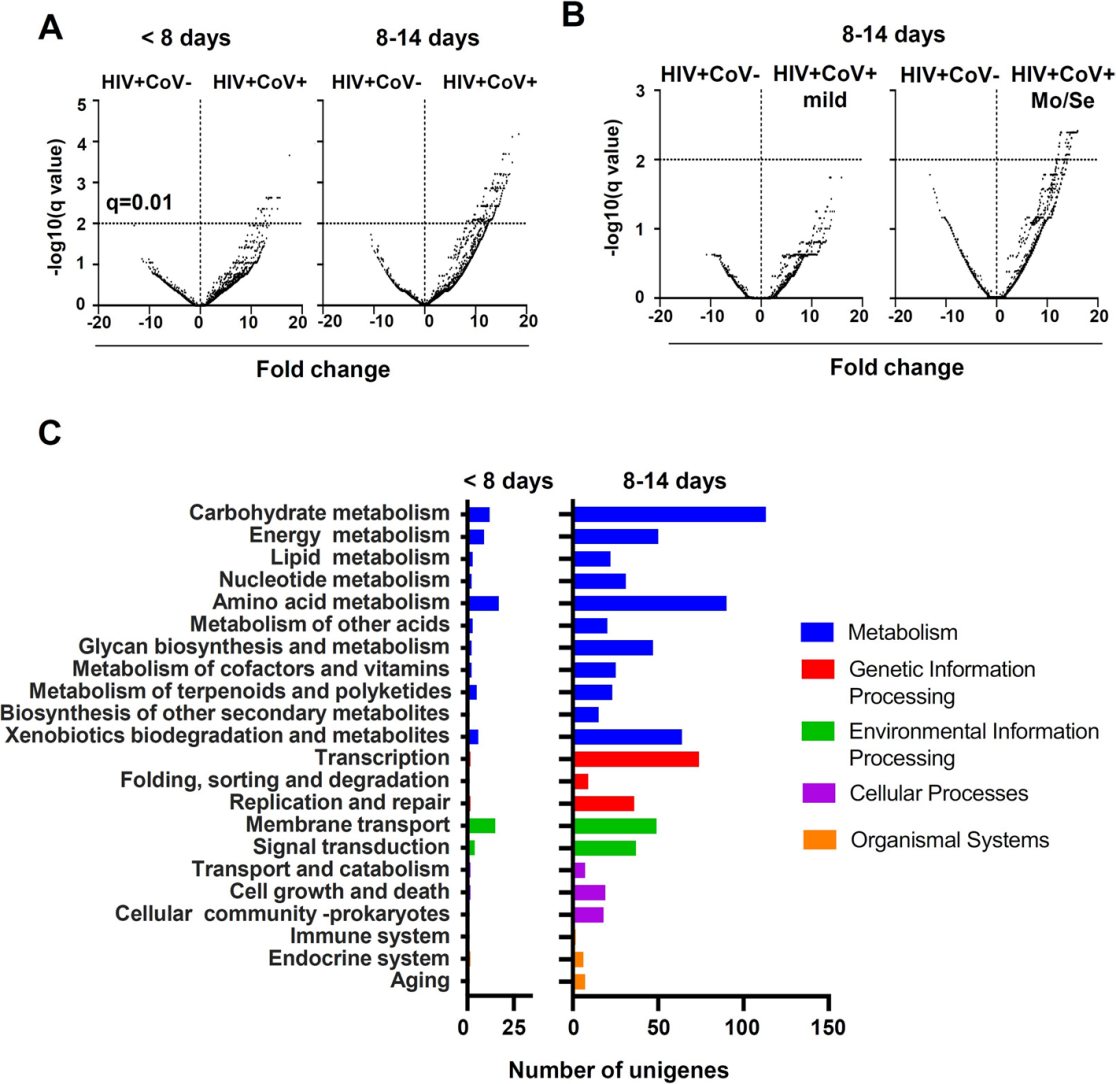
Comparison of KEGG pathways of gut microbiota-derived genes compared between SARS-CoV-2-infected and non-infected individuals in PLWH. **A**, **B** Volcano plots comparing functional genes of bacterial microbiota in PLWH within one and two weeks after COVID-19 onset with that of non-infected individuals (**A**) or classified by severity of disease and compared with non-infected individuals (**B**). **C** KEGG pathways with statistically significant differences (q < 0.01) between PLWH infected and non-infected with SARS-CoV-2 are indicated in the bar graph. < 8 days: within 7 days of COVID-19 onset; 8–14 days: 8–14 days after COVID-19 onset

To understand the intestinal environment caused by the bacterial microbiota alterations as a result of SARS-CoV-2 infection, we predicted its functionality based on sequence information obtained from 16S rRNA analysis. The predicted gene functions of the bacterial microbiota were fitted to the KEGG pathway by PICRUSt2 analysis, and pathways with significant differences between PLWH-CoV and PLWH were identified. Several enhanced gene functions were observed in PLWH-CoV, starting immediately after the onset of SARS-CoV-2 infection, and extending to the second week (q < 0.01) (Fig. 4A). Compared to PLWH without SARS-CoV-2 infection, PLWH-CoV had increased metabolic activity, mainly in three metabolic domains: carbohydrate, amino acid, and xenobiotic biodegradation (Fig. 4C). In addition, transcription and membrane transport were also enhanced in PLWH-CoV. Enrichment of these genes was observed in patients with moderate and severe disease compared to those with mild disease (Fig. 4B). In contrast, the previously reported metadata of the microbiota of SARS-CoV-2-infected and non-infected general individuals were analyzed for changes in the gene function of the bacteria (Supplementary Fig. 4A). The results showed that SARS-CoV-2 infection increased the function of several genes during the first 2 weeks of the onset of infection, but to a lesser extent than in PLWH-CoV. The genes that showed changes were mainly metabolism-related genes (Supplementary Fig. 4B).

## Discussion

This study aimed to assess the association between the pathophysiology of SARS-CoV-2 infection and the gut bacterial microbiota in PLWH, who are considered immunologically vulnerable and have a relatively high risk of severe COVID-19 due to comorbidities and other factors. The diversity of the gut microbiota of PLWH infected with SARS-CoV-2 co-infection, whose median CD4 + T cell count was 671 cells/µl before infection, was not significantly different from that of the HC or PLWH without SARS-CoV-2 infection, longer than one month after the acute phase. However, the observed dysbiosis with a decrease in SCFA-producing bacteria persisted from early onset persisted until at least one month after the onset of COVID-19, and sometimes persisted after recovery. The diversity of the gut microbiota correlated with the severity of COVID-19 disease and the dysbiosis may have contributed to the pathogenesis of PASC.

The decrease in CD4 + T cell levels after COVID-19 onset was greater in patients with moderate-to-severe disease than in those with mild disease. This observation is consistent with the lymphopenia previously reported in many patients with SARS-CoV-2 infections as previously shown [37]. In contrast, correlation analysis between disease severity and intestinal microbiota diversity showed that the increase in the Observed OTU and Shannon index were less in patients with moderate and severe disease than in patients with mild disease. This change appears to be characteristic of PLWH, based on previous observations that there was no clear change in bacterial microbiota diversity in SARS-CoV-2 patients in the general Japanese population [32]. In addition, the beta diversity analysis of the bacterial microbiota in PLWH-CoV compared with that of PLWH suggests a possible trend toward an increase in prevalence of opportunistic pathogens. This observation is consistent with the finding of previously reports of SARS-CoV-2 infections in the general population [33].

We followed the changes in bacterial microbiota over time on a weekly basis, using the onset of COVID-19 as a baseline, and observed a lack of beneficial symbiotic bacteria and an increase in opportunistic pathogens in PLWH, as dysbiosis occurred early after the onset of COVID-19. A decrease in the bacterial microbiota of the Clostridia class, such as *Anaerostipes* and *Agathobacter*, which are SCFA-producing bacteria, was observed from immediately after the onset and persisted for more than one month after clinical remission. This observation is consistent with the observed tendency toward COVID-19-associated dysbiosis reported in many studies including our previous analyses, regardless of the patient’s HIV infection status [32, 39, 40]. Furthermore, *Fusicatenibacter*, an SCFA-producing bacterium that was shown to be decreased in PLWH-CoV, is known to be decreased in inflammatory bowel disease [41]. SCFA provide an effective barrier of the intestinal epithelium, and a long-term decrease in SCFA-producing bacteria may increase the risk of systemic inflammation and metabolic disadvantages [42–45]. In PLWH, the presence of comorbidities also tended to accelerate the reduction in good bacteria, mainly SCFA-producing bacteria, and worsen COVID-19 severity. Interestingly, PLWH-CoV with moderate or severe disease tended to have worse recovery of SCFA-producing bacteria than those with mild disease, although the difference was not statistically significant. Given the tendency of a delayed recovery of the intestinal environment observed in PLWH infected with HAV [29], PLWH may tend to have a delayed recovery of the microbiota that has been altered by SARS-CoV-2 infection.

In contrast, with respect to opportunistic bacteria, *Eggerthella*, which was enriched in PLWH-CoV in the early stages after disease onset, has been reported to cause colitis and bacteremia [46, 47]. The levels of potentially pathogenic bacterial species such as Enterobacteriaceae, particularly *Escherichia-Shigella*, were also increased after 1 month after onset, and this increase was associated with body weight. In addition, a recent report found that patients with fatal COVID-19 had a higher proportion of proteobacteria phylum in their feces [48]. In our previous analysis of the gut microbiota of patients with SARS-CoV-2 infection without HIV co-infection, we observed a consistent increase in the relative abundance of *Eggerthella* from the onset, and a transient increase in *Escherichia-Shigella* was observed only one week after the onset [32]. Overall, these observations suggest that in PLWH, dysbiosis of the gut microbiota associated with SARS-CoV-2 infection leads to an environment that is more prone to inflammation and related exacerbations such as bacteremia.

In the predicted gene function of the gut microbiota of PLWH-CoV shown in the PICRUSt2 analysis, an enrichment of the metabolic pathways was observed. This observation was more pronounced than the results of functional gene prediction of the microbiota of non-PLWH SARS-CoV-2-infected individuals and may be attributed to differences in the microbiota profile. Although this effect is currently unknown, secondary metabolites released by the bacteria in a dysbiosis environment with a decrease in SCFA-producing bacteria may affect the severity of disease [49]. Further research is required to analyze these secondary metabolites as this could lead to a better understanding of the effects on the intestinal tract, including inflammation [50]. In addition, the results show that the xenobiotic biodegradation metabolism was enriched in PLWH-CoV. Although further analysis is needed to determine the significance of this bacterial pathway enrichment in PLWH who continue ART, attention should be paid to the possibility of increasing drug-resistant bacteria and reduced effectiveness of antiviral drugs.

In this study, an increase in Enterobacteriaceae and *Escherichia-Shigella* and a decrease in *Bifidobacterium* was observed in two PLWH recovering from SARS-CoV-2 infection with subjective symptoms of PASC. In previous reports, a decrease in butyrate-producing bacteria and a decrease in *Bifidobacterium* were reported in PLWH with SARS-CoV-2 infection who experienced PASC symptoms 6 months after the onset of illness [34]. Because the short-chain fatty acids produced by these bacteria are known to enhance intestinal immunity [51], although it is not certain at this time, intestinal immunity may be involved in the development of PASC. Previous reports supporting this idea have shown that in addition to a decrease in short-chain fatty acid-producing bacteria, enrichment of other bacterial groups, such as the Gammaproteobacteria class to which the Enterobacteriaceae belong, is associated with inflammatory bowel disease [52, 53]. In addition, intestinal inflammation, and expression of proinflammatory cytokines that disrupt the integrity of the intestinal barrier are associated with an increase in *Escherichia-Shigella* in the gut [54, 55]. Overall, these reports suggest that PASC in PLWH may be influenced by a negative synergistic effect of dysbiosis and intestinal inflammation.

Several limitations of this study should be acknowledged. First, we analyzed samples from only two PLWH with PASC, and long-term follow-up of further cases is needed to provide more detailed data. Second, the limited number of samples analyzed in this study would require validation with a larger number of samples to generalize the conclusions—for example, the present study did not analyze the bacterial microbiota in terms of diet, habits, steroid or PPI use. The impact of different SARS-CoV-2 variants on the gut microbiota was also not analyzed. Data on the gut microbiota before infection would allow a more detailed analysis of the changes in the gut microbiota during the acute phase and would be useful in the interpretation of the data over time. Third, the question of how the observed changes in diversity after infection affect the severity of disease caused by SARS-CoV-2 infection has not been resolved in this study.

Future research directions include a detailed understanding of the changes in bacterial microbiota diversity and analysis of bacterial metabolites and secretions such as extracellular vesicles during post-onset dysbiosis, which will help us to better understand the disease at the molecular level [56]. The findings may establish methodologies for therapeutic intervention, including control of the gut microbiota, to avoid the risk of severe disease. The ART regimen, particularly integrase strand transfer inhibitors (INSTIs), affects the composition of the gut microbiota in PLWH [57, 58], and among those taking ART containing INSTIs the recovery of bacterial translocations, and the plasma sCD14 level is the same as that of healthy individuals [58]. Further analysis of the effect of ART regimens on disease progression and outcomes in COVID-19 is required. In summary, the abrupt abnormalities in the gut microbiota associated with SARS-CoV-2 infection observed in PLWH in this study indicated an intestinal environment that is likely to trigger disease progression. However, data on the relationship between the composition of the bacterial microbiota and the development of SARS-CoV-2 are scarce. Clarification of this relationship may lead to the planning and development of new prevention and treatment strategies against SARS-CoV-2. Therefore, detailed monitoring of bacterial microbiota profiles would help to understand the molecular basis of COVID-19 severity in immunocompromised patients.

## Materials and methods

### Subject recruitment and sample collection

We collected stool and blood samples from 12 people living with HIV (PLWH) with SARS-CoV-2 infection who were hospitalized at the University of Tokyo Institute of Medical Science Hospital. Subjects who were co-infected with hepatitis virus in addition to HIV infection were excluded from the analysis. The patient was classified as mild disease if there were no signs of pneumonia on CT images [59]. The patient was classified as a moderate disease if there were signs of pneumonia on CT images with fever, respiratory symptoms, and oxygen saturation greater than 94% [59]. Of the 12 patients, 3 were also using steroids at the time of admission. Blood and stool samples were collected from patients during hospitalization. The seven patients who were discharged from the hospital provided stool specimens at the follow-up date (1 month or later after the onset of the disease). Stool samples were collected and provided by the patients themselves during hospitalization and stool specimens were brought to the hospital by the patients after discharge. Blood and stool specimens were immediately transported to a laboratory near the hospital. Stool specimens were stored at − 80 °C before DNA preparation. The plasma fraction of the blood specimens was stored at − 80 °C. For the control group, we used stool samples from 19 healthy subjects and 25 non-SARS-CoV-2 infected PLWH recruited. Healthy Cohort (HC) who reported pre-existing medical conditions were excluded from the study. All participants (HC and PLWH) as the control group had not been vaccinated against SARS-CoV-2, and their medical histories are listed in Table 2.

### DNA extraction, amplification, and 16S rRNA gene sequencing

We extracted DNA from the fecal sample-derived bacterial fraction, as previously described [20]. In brief, human fecal samples were stored at − 80 °C immediately after collection. One gram of human fecal sample was homogenized in 3 ml of SM-plus buffer (100 mM NaCl, 50 mM Tris-HCl (pH 7.4), 8 mM MgSO4⋅7H_2_O, 5 mM CaCl⋅2H_2_O, 0.01% (w/v) gelatin in distilled water) with vortex homogenization by mixing and filtered through a 100 μm cell strainer. The residue on the cell strainer was washed twice with 3 ml of SM-plus buffer, and the filtrate was centrifuged at 6,000 × g for 5 min. The supernatant was discarded, and the remaining pellet was used for the bacterial fraction. The bacterial cell wall was lysed with bacteriolysis enzyme, and bacterial DNA was extracted by phenol-chloroform extraction and ethanol extraction for protein removal and purification. The 16S rRNA gene libraries were prepared according to the 16S Metagenomics Sequencing Library Preparation guide (Part #15,044,223 Rev. B; Illumina, San Diego, CA, USA). Briefly, the hypervariable V3–V4 region of the 16S rRNA gene was amplified using specific primers: forward (5′-ACACGACGCTCTTCCGATCTCCTACGGGNGGCWGCAG-3′) and reverse (5′-GACGTGTGCTCTTCCGATCTGACTACHVGGGTATCTAATCC-3′), comprising Illumina adapter overhang nucleotide sequences (underlined) [60]. Next, adapter ligation for polymerase chain reaction (PCR) amplicons was performed using NEBNext Multiplex Oligos for Illumina (Dual Index Primers Set 1; New England Biolabs, Ipswich, MA, USA). Sequencing was performed on the Illumina MiSeq system (Illumina) using the MiSeq Reagent Kit v3 (600-cycle) with a 15% PhiX (Illumina) spike-in.

### Sequencing and statistical analyses

Sequences were quality filtered, denoised, and analyzed using Quantitative Insights Into Microbial Ecology 2 (QIIME 2 version 2021.2) as previously reported [61]. In brief, DADA2 was used to denoise the paired-end reads into amplicon sequence variants [62]. A bacterial taxonomic classification was assigned to the resulting amplicon sequence variants against the SILVA database (release 132) [63]. This was trimmed to the V3–V4 region of the 16S rRNA gene using a naïve Bayesian classification method [64]. The Kruskal-Wallis test was used for statistical analysis of alpha diversity (Shannon index) and analyzed with QIIME2 software (cut-off *p*-value < 0.05). OTU tables were aligned to an equal sampling depth of 10,000 per sample by alpha-rarefaction analysis to avoid bias caused by differences in sequence depth. Data were pre-processed as described in ANCOM-II to remove low-abundance or rare taxa before differential presence ratio analysis [65]. Unweighted and weighted uniFrac distances for β-diversity were displayed in a distance matrix computed by QUIIME2. Permutational multivariate analysis of variance (PERMANOVA) was used to analyze the β-diversity and complexity of bacterial communities in the samples. PICRUSt2 was used to predict the microbial content from the sequence information of each sample and to make functional predictions based on the genes possessed by the bacteria [66]. The Kyoto Encyclopedia of Genes and Genomes (KEGG) database, which contains data on compounds, reactions, enzymes, and metabolic pathways that have been experimentally validated and reported in the scientific literature, was used to examine the predicted metabolic networks of organisms [67]. All genus-level differential abundances of bacteria were tested using the Mann-Whitney U test. The significance of all tests was *p* < 0.05 or False Discovery Rate corrected *p* < 0.05 (two-tailed). Pearson’s chi square test was used for bivariate analyses. All data were statistically compared using GraphPad Prism v9. Metagenomic profile statistical analysis was performed using the LEfSe method was used to identify differentially abundant taxa [68]. To validate the output generated from LEfSe, we analyzed the compositional microbiome with bias correction (adjustment for BMI) using multivariate analysis with linear models (MaAsLin)2 [69]. The default values of the parameters were used in this analysis (minimal prevalence: 0.1, max significance: 0.25, and normalization: TSS). The R program (version 4.3.2, https://www.r-project.org) was used for the MaAsLin2 analysis. The analysis did not involve the centered-log ratio or any other method for normalizing or transforming relative bacterial abundance data.

### Supplementary Information

**Additional file 1: Supplementary Fig. 1.** Longitudinal changing of plasma CD4 T + cell count in PLWH from 1 years before to 6 month after COVID-19 disease onset.

**Additional file 2: Supplementary Fig. 2.** Impact of medical history and treatment background of PLWH on changes in gut microbiota after SARS-CoV-2 infection (A) Comparison of gut microbiota in PLWH-CoV with and without comorbidities 8–14 days after SARS-CoV-2 infection (B) Length of ART treatment compared by severity of disease (mild vs. moderate/severe) (C) Longitudinal plot of Observed OTUs (blue) and Shannon index (red) in patients who did not suffer from PASC symptoms. ns: not significant.

**Additional file 3: Supplementary Fig. 3.** Major genus level of bacterial changes (mean values) in PLWH-CoV classified by severity of Disease. SCFA: short-chain fatty acid, < 8 days: within 7 days of COVID-19 onset; 8–14 days: 8–14 days after COVID-19 onset, g: genus.

**Additional file 4: Supplementary Fig. 4.** KEGG pathway comparison predicted from bacterial-derived gene enrichment between SARS-CoV-2 Infection and non-infection in healthy subjects. (A) Volcano plots of bacterial microbiota in healthy subjects compared to uninfected subjects within 1 and 2 weeks from COVID-19 onset. (B) KEGG pathways with statistically significant differences (q < 0.01) among healthy subjects infected or uninfected with SARS-CoV-2 are shown in the bar graph. < 8 days: within 7 days of COVID-19 onset; 8–14 days: 8–14 days after COVID-19 onset.

**Additional file 5: Supplementary Table 1.** Pairwise permanova results in unweighted and weighted Unifrac significance. **Supplementary Table 2. **Overview of the demographic and clinical characteristics of participants in this study

## Data Availability

The datasets generated and/or analysed during the current study are available in the DNA Data Bank of Japan (DDBJ), [https://ddbj.nig.ac.jp/search/en; accession number: DRA012374 and DRA015910]
