# Supplementary figures and images for: Association of gut microbiota with the pathogenesis of SARS-CoV-2 Infection in people living with HIV
Source: BMC Microbiol. 2024 Jan 3;24:6. doi: 10.1186/s12866-023-03157-5 (PMC10763188; doi:10.1186/s12866-023-03157-5)

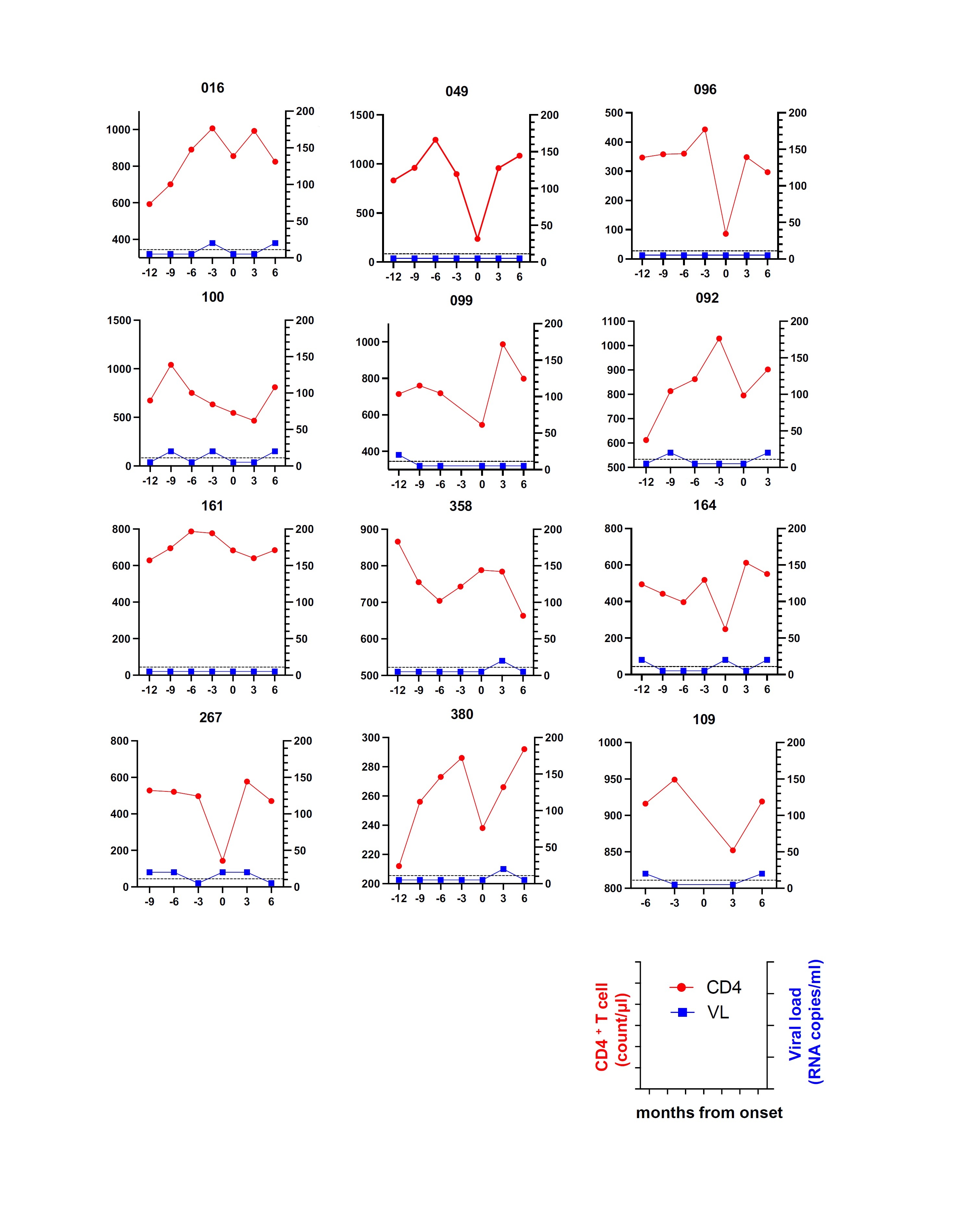

Supplement: Supplementary file 1 — Additional file 1: Supplementary Fig. 1. Longitudinal changing of plasma CD4 T + cell count in PLWH from 1 years before to 6 month after COVID-19 disease onset. [file 12866_2023_3157_MOESM1_ESM.jpg]

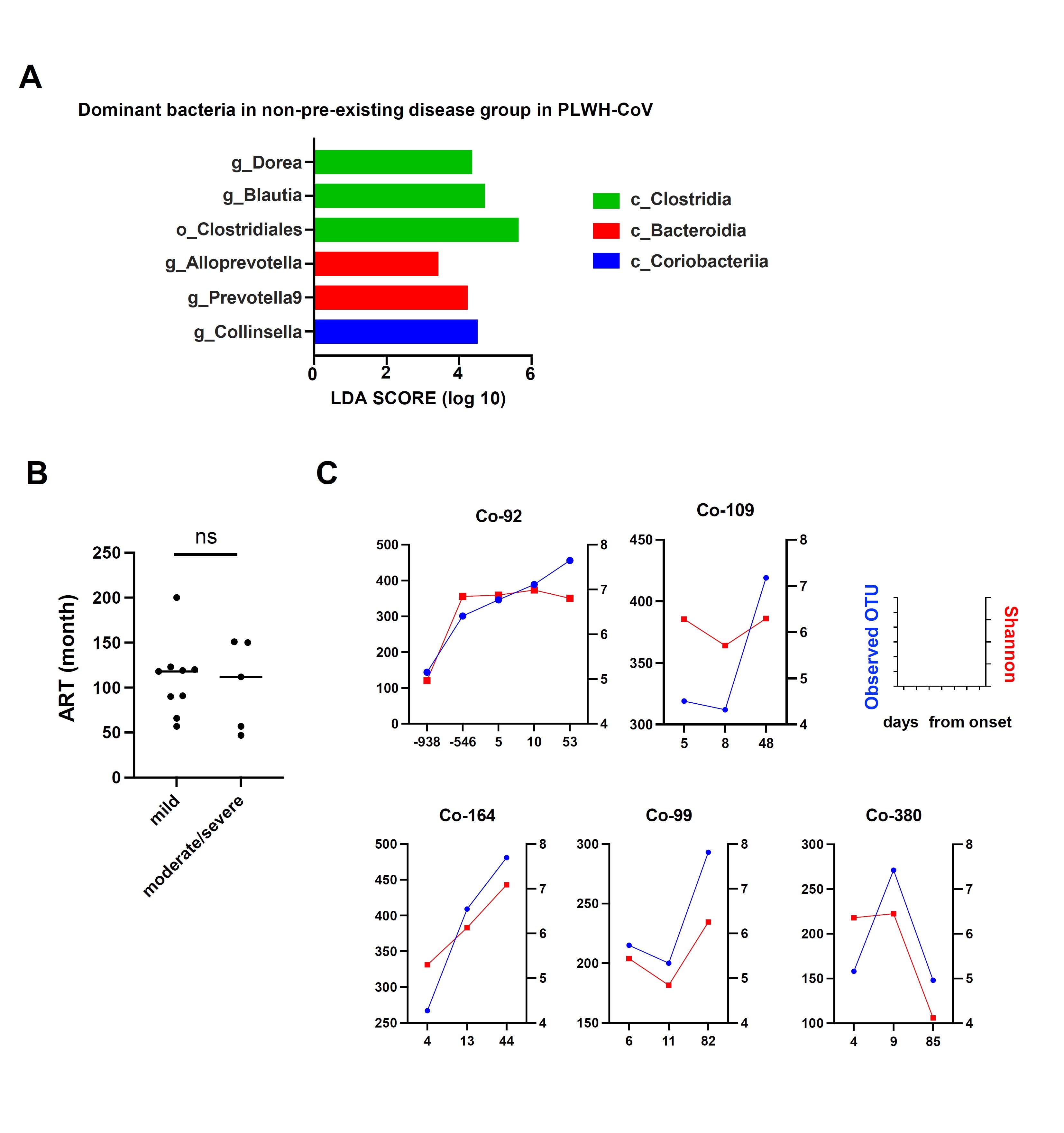

Supplement: Supplementary file 2 — Additional file 2: Supplementary Fig. 2. Impact of medical history and treatment background of PLWH on changes in gut microbiota after SARS-CoV-2 infection (A) Comparison of gut microbiota in PLWH-CoV with and without comorbidities 8–14 days after SARS-CoV-2 infection (B) Length of ART treatment compared by severity of disease (mild vs. moderate/severe) (C) Longitudinal plot of Observed OTUs (blue) and Shannon index (red) in patients who did not suffer from PASC symptoms. ns: not significant. [file 12866_2023_3157_MOESM2_ESM.jpg]

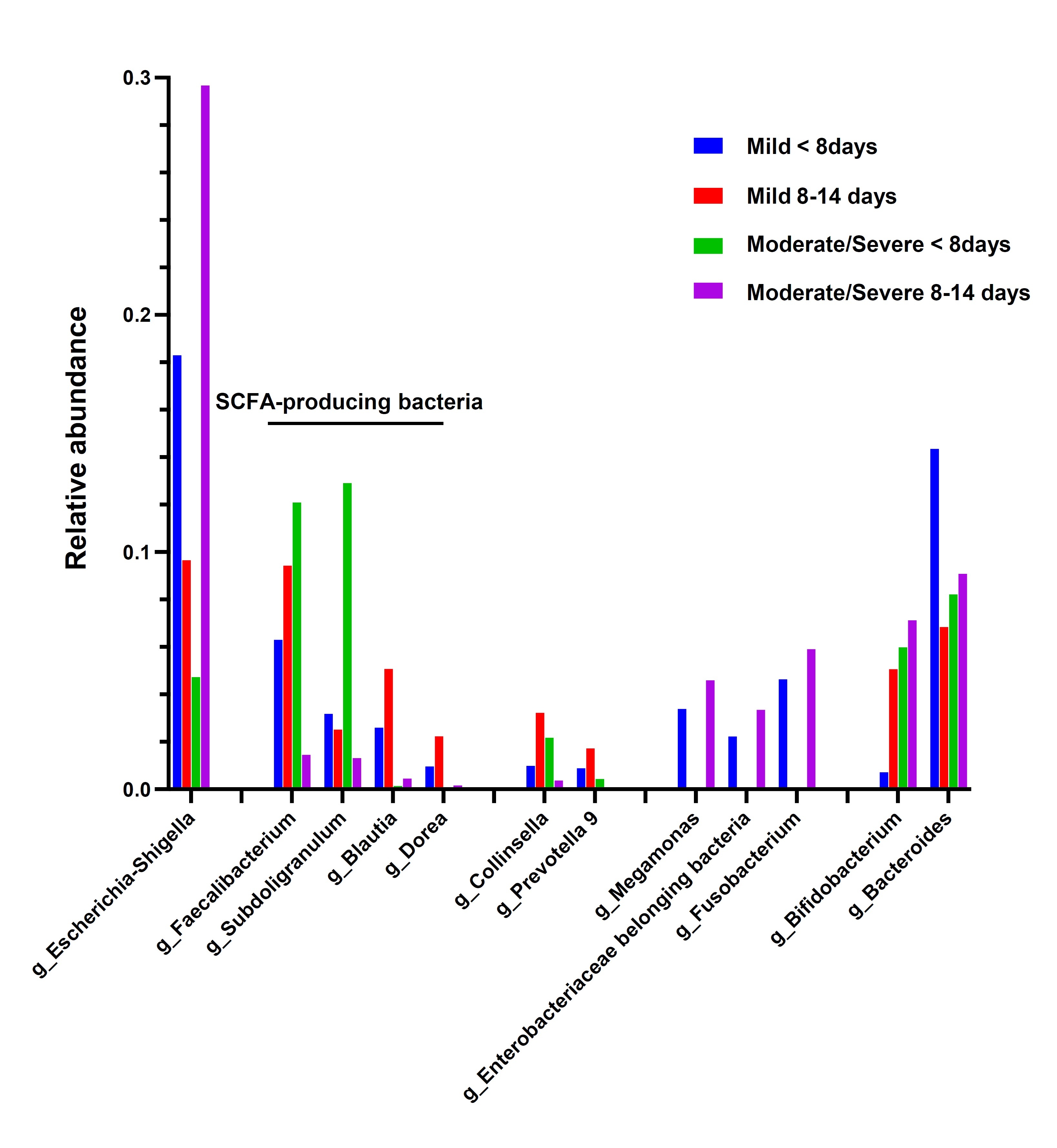

Supplement: Supplementary file 3 — Additional file 3: Supplementary Fig. 3. Major genus level of bacterial changes (mean values) in PLWH-CoV classified by severity of Disease. SCFA: short-chain fatty acid, < 8 days: within 7 days of COVID-19 onset; 8–14 days: 8–14 days after COVID-19 onset, g: genus. [file 12866_2023_3157_MOESM3_ESM.jpg]

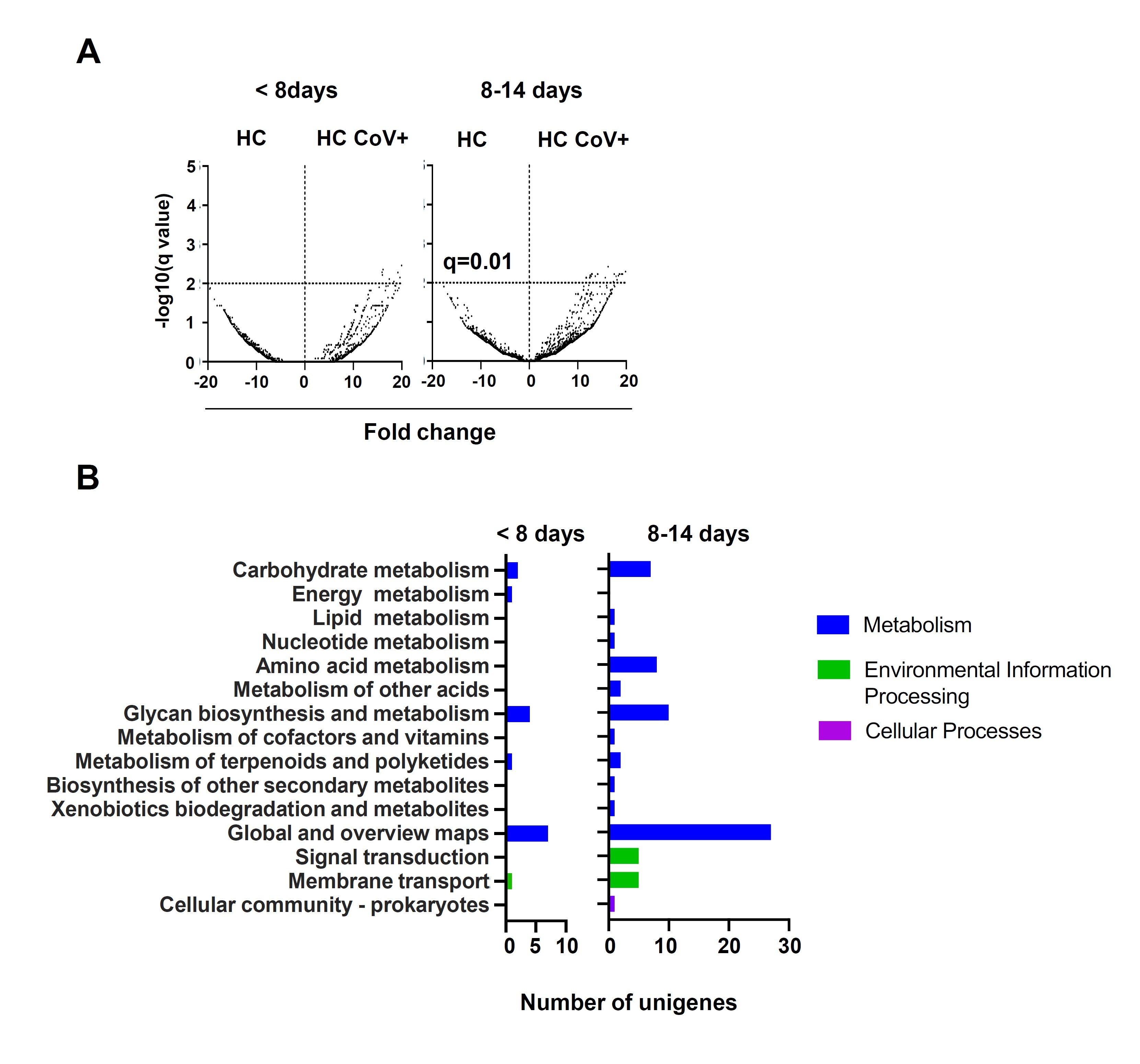

Supplement: Supplementary file 4 — Additional file 4: Supplementary Fig. 4. KEGG pathway comparison predicted from bacterial-derived gene enrichment between SARS-CoV-2 Infection and non-infection in healthy subjects. (A) Volcano plots of bacterial microbiota in healthy subjects compared to uninfected subjects within 1 and 2 weeks from COVID-19 onset. (B) KEGG pathways with statistically significant differences (q < 0.01) among healthy subjects infected or uninfected with SARS-CoV-2 are shown in the bar graph. < 8 days: within 7 days of COVID-19 onset; 8–14 days: 8–14 days after COVID-19 onset. [file 12866_2023_3157_MOESM4_ESM.jpg]
